# Early Postnatal Cardiomyocyte Proliferation Requires High Oxidative Energy Metabolism

**DOI:** 10.1038/s41598-017-15656-3

**Published:** 2017-11-13

**Authors:** Ana Elisa Teófilo Saturi de Carvalho, Vinícius Bassaneze, Maria Fernanda Forni, Aline Alfonso Keusseyan, Alicia Juliana Kowaltowski, José Eduardo Krieger

**Affiliations:** 1 Laboratory of Genetics and Molecular Cardiology/LIM 13, Heart Institute (InCor- HCFMUSP), University of São Paulo Medical School, São Paulo, Brazil; 2 Departamento de Bioquímica, Instituto de Química, Universidade de São Paulo, São Paulo, Brazil

## Abstract

Cardiac energy metabolism must cope with early postnatal changes in tissue oxygen tensions, hemodynamics, and cell proliferation to sustain development. Here, we tested the hypothesis that proliferating neonatal cardiomyocytes are dependent on high oxidative energy metabolism. We show that energy-related gene expression does not correlate with functional oxidative measurements in the developing heart. Gene expression analysis suggests a gradual overall upregulation of oxidative-related genes and pathways, whereas functional assessment in both cardiac tissue and cultured cardiomyocytes indicated that oxidative metabolism decreases between the first and seventh days after birth. Cardiomyocyte extracellular flux analysis indicated that the decrease in oxidative metabolism between the first and seventh days after birth was mostly related to lower rates of ATP-linked mitochondrial respiration, suggesting that overall energetic demands decrease during this period. In parallel, the proliferation rate was higher for early cardiomyocytes. Furthermore, *in vitro* nonlethal chemical inhibition of mitochondrial respiration reduced the proliferative capacity of early cardiomyocytes, indicating a high energy demand to sustain cardiomyocyte proliferation. Altogether, we provide evidence that early postnatal cardiomyocyte proliferative capacity correlates with high oxidative energy metabolism. The energy requirement decreases as the proliferation ceases in the following days, and both oxidative-dependent metabolism and anaerobic glycolysis subside.

## Introduction

The neonatal cardiac tissue dilplay high tissue proliferation and differentiation, but these processes subside within the first week after birth^1,2^. Indeed, neonatal cardiomyocytes proliferate well up to the first postnatal week^3^. After this period, even though there is DNA synthesis, it is mostly associated with multinucleation. In young animals, cardiomyocytes turnover rate is around 1% per year, and this decreases with age^4,5^. DNA synthesis is one of the steps in this process and can lead to multinucleation in cardiac myocytes. There are different methodological approaches to assay them^6,7^.

Proliferation and differentiation are both energy-demanding processes, and, as a result, changes in energy metabolism are expected as the neonatal cardiac properties rapidly evolve within this short postnatal period. Indeed, other tissues undergoing rapid expansion, such as fetal liver primitive hematopoietic stem cells, require an efficient energy source to fuel cellular expansion^8^. Furthermore, mesenchymal stem cell differentiation also involves increased oxidative metabolic capacity that is specific for the type of differentiation. Osteocytes and adipocytes require enhanced respiratory capacity, whereas commitment to chondrogenesis leads to a loss of respiratory capacity. Blocking mitochondrial plasticity in these models prevents differentiation, demonstrating that changes in oxidative metabolism are required for this process^9^.

In the heart, postnatal development is believed to involve a shift from glycolytic fermentation to oxidative phosphorylation (reviewed in^10^). In perfused rabbit hearts, glycolytic rates were found to decrease between the 1^st^ and 7^th^ postnatal days, with a concomitant increase in fatty acid oxidation^11^. These results are consistent with a shift from fermentative to oxidative metabolism; however, lactate oxidation rates remain unchanged at both time points, which is not consistent with this view^11^. Further support for the concept that heart development following birth involves a shift to more oxidative metabolism comes from a PGC-1α/β mouse model^12^, which lacks the transcriptional co-activators responsible for promoting mitochondrial biogenesis and presents postnatal heart maturation defects, dying shortly after birth. However, PCG-1 family proteins display other functions that may contribute toward the abnormalities, including the regulation of fatty acid oxidation. More recently, Puente *et al*. elegantly demonstrated that postnatal heart maturation involves enhanced oxidative tissue damage and that prevention of this damage by antioxidants or hypoxic conditions maintain the proliferative capacity of neonatal cardiomyocytes^13^. Based on these results and the knowledge that mitochondrial mass increases during postnatal cardiac development^14^, Puente *et al*. proposed that, after birth, atmospheric oxygen promotes an increase in mitochondrial oxidative activity resulting in enhanced production of reactive oxygen species. Interestingly, however, although markers of mitochondrial mass have been quantified^13,14^, no direct functional studies comparing oxygen consumption rates in hearts with high proliferation capacity (P1) and low proliferation capacity (P7) have been conducted. In this study, we measured respiratory activity in neonatal rat hearts and found, quite surprisingly, that oxygen consumption activity does not correlate well with expression patterns of oxidative genes, and decreases during maturation. Furthermore, we demonstrate that maintaining high oxygen consumption capacity is necessary to sustain the proliferative capability of neonatal cardiomyocytes.

## Results

### Higher glycolysis and oxygen consumption in the P1 heart

**Figure 1:**
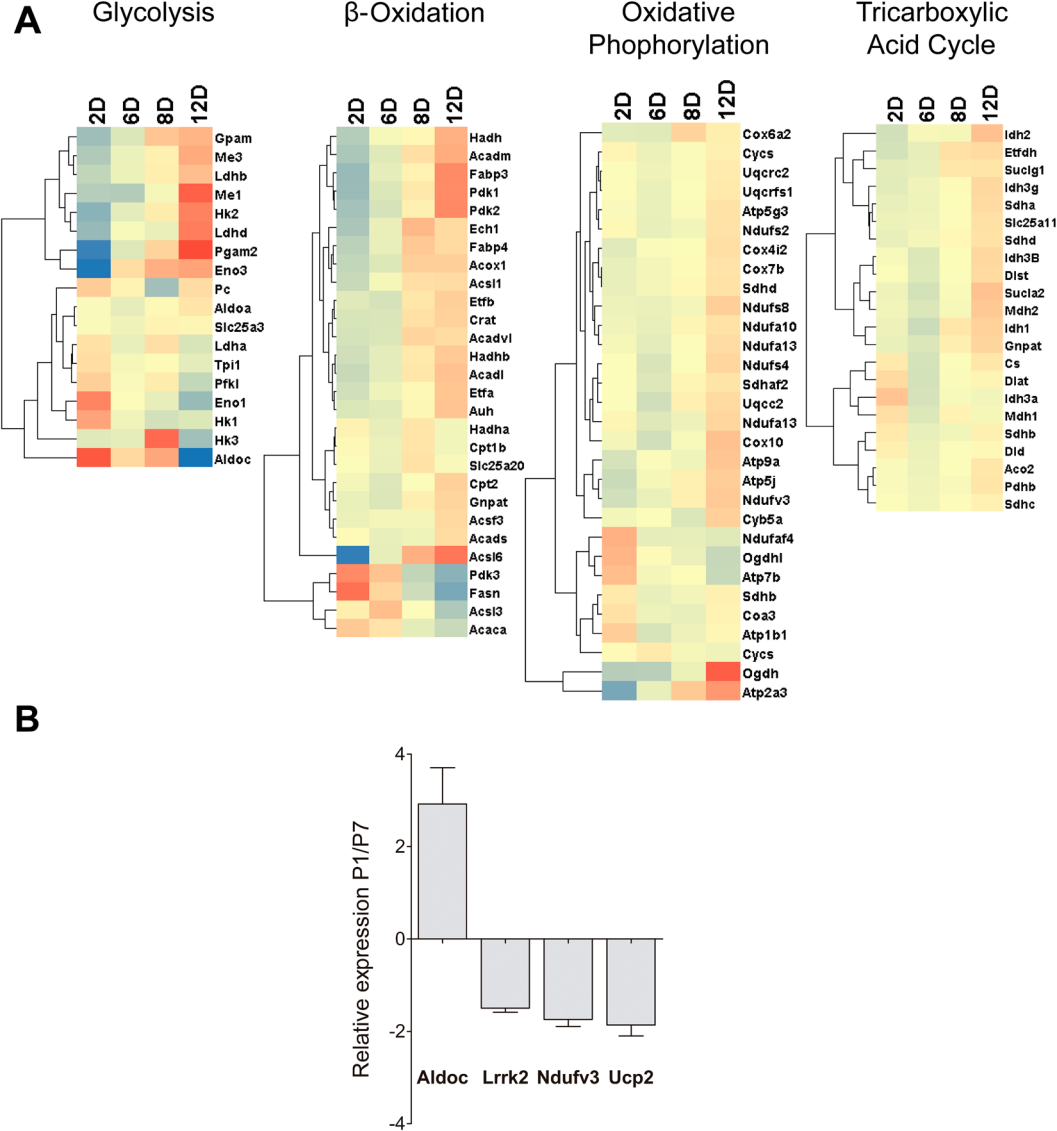
Gene expression analysis. (**A**) Heat map showing gene expression assayed by RNASeq. The heat maps show gene clusters involved in glycolysis, β-oxidation, oxidative phosphorylation and the tricarboxylic acid cycle in rat hearts from 2, 6, 8, and 12 days after birth (n = 6 for both). (**B**) Quantitative RT-PCR analysis of genes involved in cell metabolism. Bars indicate the relative expression (2^−ΔΔct^) for P1 versus P7 hearts (n = 6 for both) of Aldolase c (a glycolysis enzyme) and mitochondrial Lrrk2, Ndufv3, and Ucp2; t-test, p < 0.05.

There is evidence that the postnatal heart becomes dependent on oxidative catabolic pathways as proliferative capacity declines, in a process possibly induced by atmospheric oxygen^13^. Indeed, we found that RNASeq expression analysis of genes involved in oxidative catabolic pathways, such as β-oxidation, the tricarboxylic acid cycle, and oxidative phosphorylation, showed a gradual overall upregulation during the postnatal period (Fig. 1A). A concomitant exchange in the expression profile of glycolytic enzymes occurred within the first days after birth. RNASeq results were confirmed by qRT-PCR experiments showing that mitochondrial genes were downregulated in P1 relative to P7, while glycolytic Aldolase c gene (Aldoc) was upregulated (Fig. 1B).

**Figure 2:**
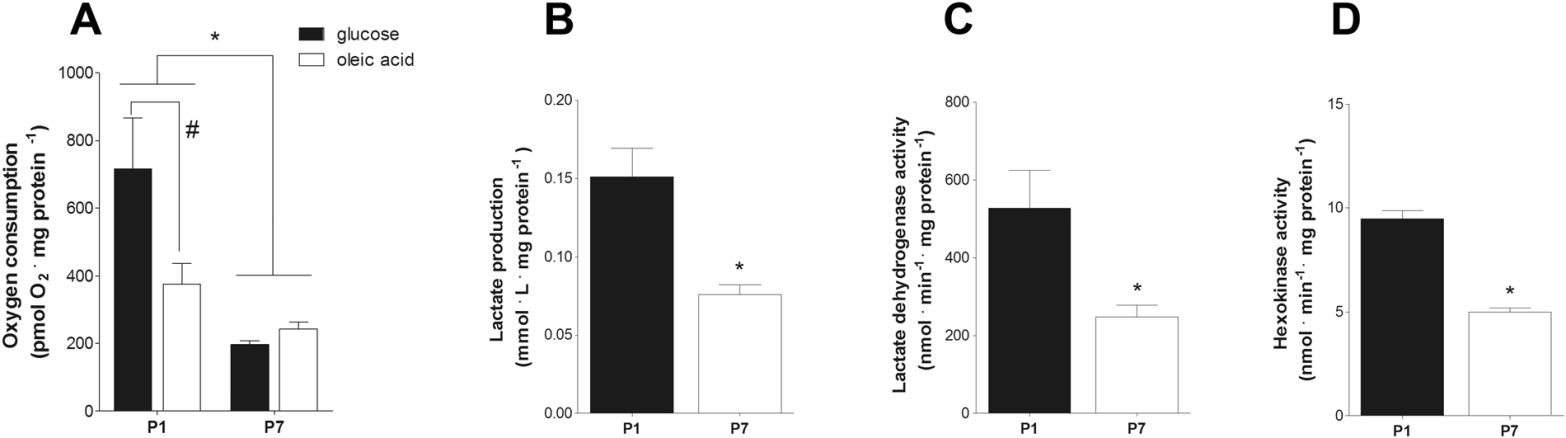
High oxygen consumption and glycolytic capacity in P1 heart tissue. (**A**) Bars indicate oxygen consumption (mean ± SEM) determined by use of an Oroboros O2k high-resolution respirometer in P1 (n = 5) and P7 (n = 6) hearts with glucose (5 mM) or oleic acid (100 µM) as substrates; two-way ANOVA, *p < 0.05 vs. P1, ^#^p < 0.05 vs. glucose in P1. (**B**) Lactate levels (mean ± SEM) measured in the oxygen consumption conditioned buffer for P1 and P7 hearts (n = 6 for both); t-test, *p < 0.05 vs. P1. (**C**) Lactate dehydrogenase activity (mean ± SEM) in P1 and P7 hearts (n = 5 for both); t-test, *p < 0.05 vs. P1. (**D**) Hexokinase activity (mean ± SEM) in P1 and P7 hearts (n = 6 for both); t-test, *p < 0.05 vs. P1.

**Figure 3:**
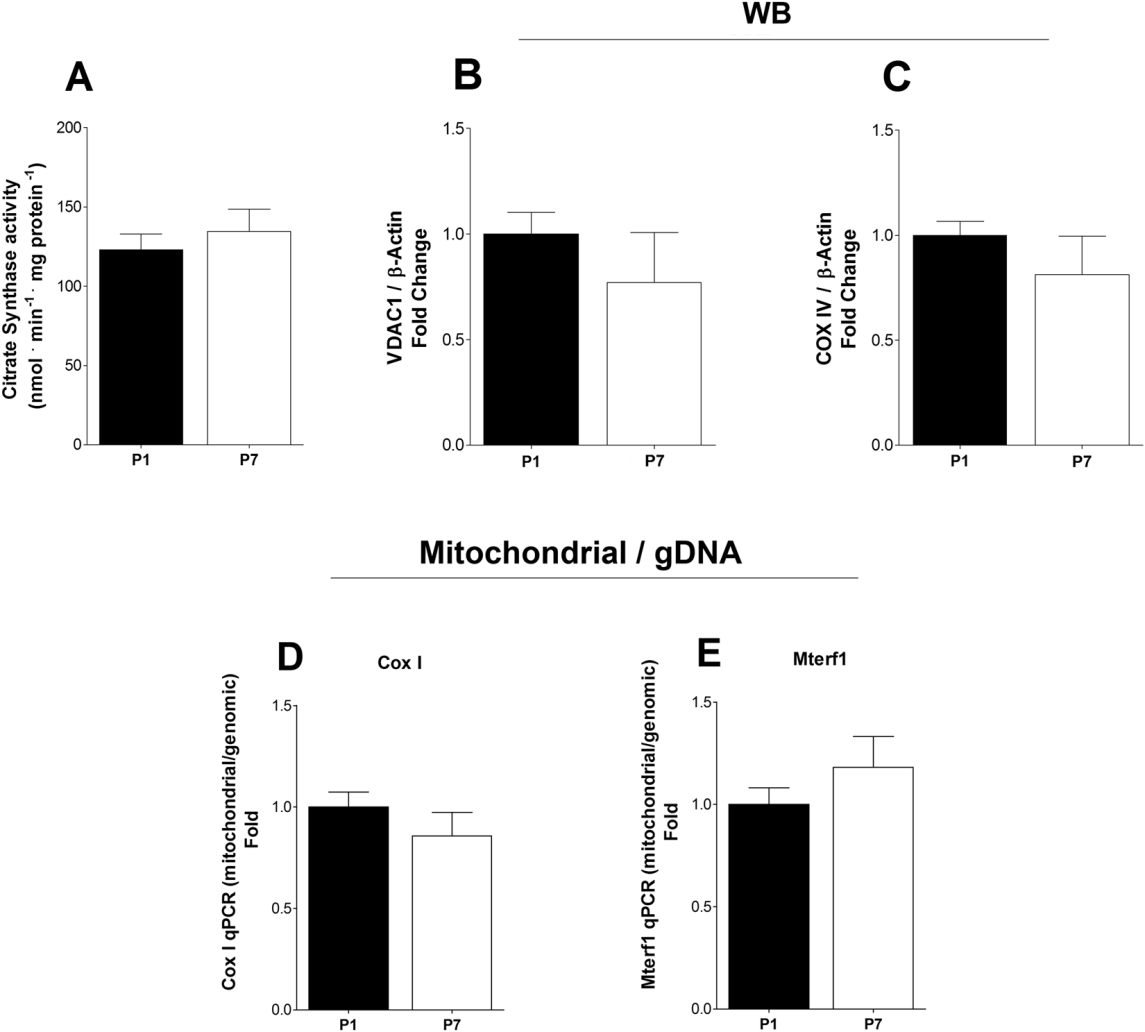
Postnatal metabolic maturation does not alter mitochondrial mass. (**A**) Citrate synthase activity (mean ± SEM) in P1 and P7 hearts (n = 6 for both); t-test, NS. (**B**) VDAC1 protein expression (mean ± SEM) in P1 and P7 hearts (n = 6 for both) measured by Western Blotting; t-test, NS. (**C**) COX IV protein expression (mean ± SEM) in P1 and P7 hearts (n = 6 for both) measured by Western Blotting; t-test, NS. (**D**) Mitochondrial Cox I and (**E**) Mterf1DNA quantification relative to genomic DNA in P1 and P7 heart tissue (n = 6 for both); t-test, NS.

Despite the widespread and robust results in gene expression patterns observed, most metabolic regulation occurs via allosteric and post-translational mechanisms^15–17^, which may not reflect gene expression values, so we functionally evaluated oxidative metabolism in P1 and P7 hearts. Tissue samples collected from P1 and P7 pups, and oxygen consumption rates, corrected for tissue protein content, were compared using high resolution respirometry^18^ using either glucose or fatty acids as substrates (Fig. 2A). Interestingly, we found that despite the upregulation of genes involved in oxidative metabolism, basal oxygen consumption rates decreased significantly between P1 and P7, with both glucose and fatty acids as substrates, although more strikingly with the former. Not only did glucose-supported oxygen consumption decrease, but the production of lactate (Fig. 2B) also dropped, indicating that both complete oxidation of glucose to CO_2_ and water through oxidative phosphorylation and fermentation of glucose to lactate dampened during postnatal maturation. Confirming that glycolytic activity decreased between P1 and P7, we found that the activity of lactate dehydrogenase (Fig. 2C) and hexokinase (Fig. 2D) decreased during this period, despite the stimulated gene expression depicted in Fig. 1A. To further characterize metabolic changes in neonatal cardiac cells, we measured mitochondrial content by quantifying markers of mitochondrial biogenesis and mass in heart tissue samples. Citrate synthase activity (Fig. 3A), VDAC and COX IV levels (Fig. 3B and C, respectively) were equal between P1 and P7 heart tissue. Similarly, mitochondrial DNA normalized by genomic DNA is equal in P1 and P7 ventricles (Fig. 3D and E) and cultured cells (Supp. Figure 1). Overall, these results highlight the limitations of using gene expression patterns to evaluate functional oxidative status in the developing heart. They further demonstrate that the postnatal period involves dampening of anaerobic glycolysis and oxidative catabolic activity, which do not involve changes in mitochondrial content.

### Enhanced maximal and basal oxygen consumption rates in P1 cardiomyocytes

**Figure 4:**
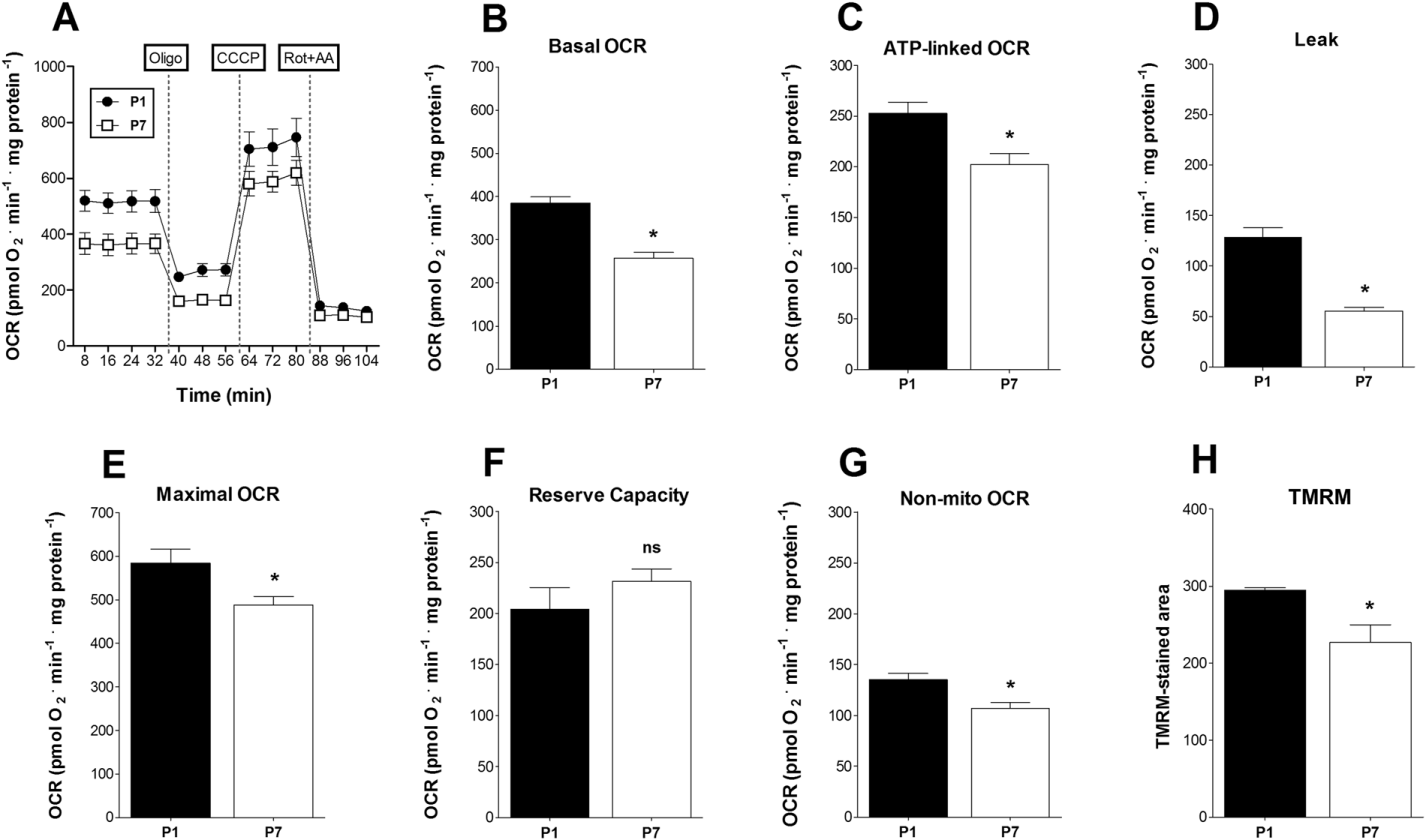
P1 cardiomyocytes have a significant mitochondrial oxidative metabolism. (**A**) Time scan measurements of real-time oxygen consumption (OCR) using a Seahorse Flux Analyzer in cardiomyocyte cultures from P1 and P7 (n = 3 for both). Panels B-H show quantitative comparisons derived from experiments (mean ± SEM), such as those in panel (A,B) Basal OCR; t-test, *p < 0.05 vs. P1. (**C**) ATP-linked OCR; t-test, *p < 0.05 vs. P1. (**D**) Proton leak; t-test, *p < 0.05 vs. P1. (**E**) Maximal OCR; t-test, *p < 0.05 vs. P1. (**F**) Reserve capacity; t-test, NS. (**G**) Non-mitochondrial; t-test, *p < 0.05 vs. P1. (**H**) TMRM-stained the cell-integrated pixel area (mean ± SEM) in cardiomyocyte cultures from P1 and P7 pups (n = 3 for both); t-test, *p < 0.05 vs. P1.

Tissue oxygen consumption allows for an evaluation of basal oxidative activity, but cannot readily determine maximal oxidative capacity or how much of this activity reflects ATP production. To assess these parameters and further confirm our results in tissue samples, we prepared P1 and P7 cardiomyocytes cultures and compared their oxygen consumption rates using extracellular flux analysis^19,20^ (Fig. 4). Figure 4A shows a typical flux analysis experiment confirming that basal respiration is higher in P1 cells (results are quantified in Fig. 4B). The addition of the ATP synthase inhibitor oligomycin allows for the determination of the respiratory rates linked to ATP production (oligomycin-inhibited) and the proton leak (oligomycin-insensitive). In both (Fig. 4C and D, respectively) the values decreased in P7 relative to P1 cardiomyocytes. A subsequent addition of the uncoupler CCCP promotes maximal respiratory rates, which were higher in P1 cells (Fig. 4E). The spare respiratory capacity (calculated as maximal respiration minus basal), often associated with protection against damaging conditions^21^, was equal in P1 and P7 cells (Fig. 4F). Finally, the addition of the electron transport inhibitors rotenone and antimycin allowed for the determination of non-mitochondrial oxygen consumption rates, which were slightly lower in P7 cells (Fig. 4G) but contributed little to overall oxygen consumption. We subtracted these rates from all other measurements. Altogether, these results confirm the findings with intact cardiac tissue, indicating that basal respiratory activity decreased between the first and seventh postnatal days, and indicate that this decrease is related to a decrease in ATP-linked oxygen consumption. Also, it shows that the maximal respiratory capacity of P1 cardiomyocytes is higher than P7. Given the surprising results of our oxygen consumption measurements, we decided to visualize the area of functional mitochondria in live cells using the membrane potential-sensitive fluorescent dye tetramethylrhodamine methyl ester (TMRM), which accumulates in mitochondria sustaining active membrane potentials. We observed that P1 cardiomyocytes displayed larger areas of active mitochondria (Fig. 4H), once again confirming that a functional decrease in mitochondrial oxidative activity occurs between P1 and P7.

### Robust oxidative metabolism is necessary for cardiomyocyte proliferation

**Figure 5:**
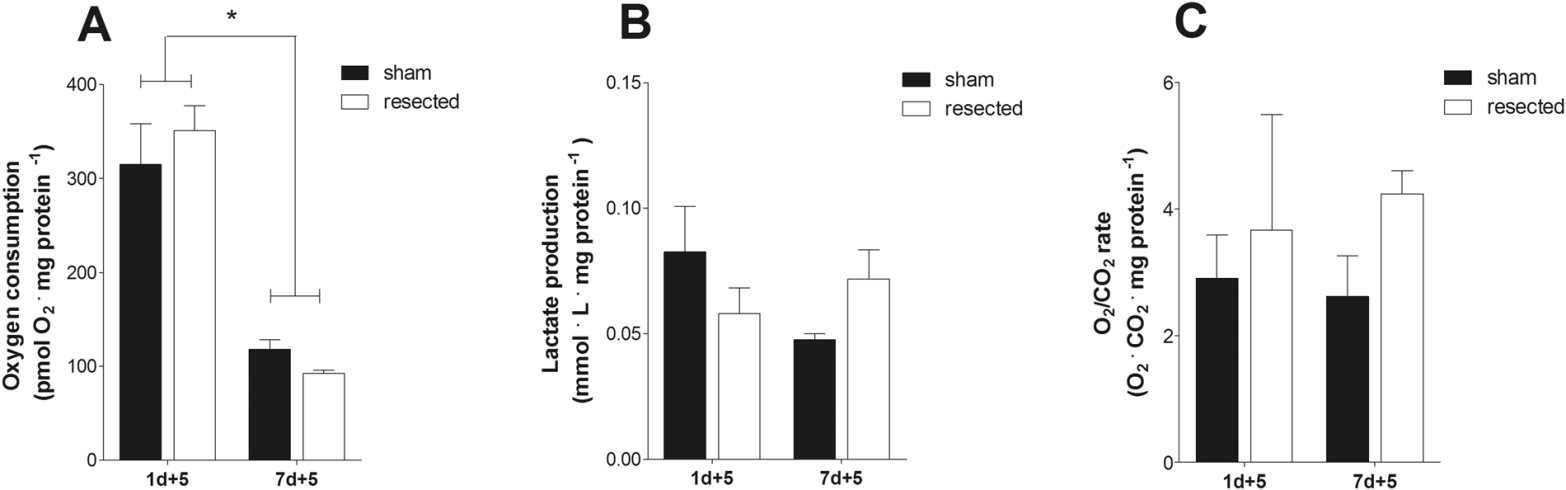
Cardiac cell proliferation does not alter tissue metabolic profiles. (**A**) Bars indicate oxygen consumption (mean ± SEM) with glucose (5 mM) measured using Oroboros O2k high-resolution respirometry in P1, P7, and sham resected neonatal hearts 5 days (+5) after the procedure (S1 + 5d, n = 8 and R1 + 5d, n = 3; S7 + 5d, n = 7 and R7 + 5d, n = 4); two-way ANOV*A*, *p < 0.05 vs. 1d + 5. (**B**) Lactate production (mean ± SEM) measured in the oxygen consumption conditioned buffer from P1 and P7 sham and resected neonatal hearts five days after the procedure (S1 + 5d, n = 8 and R1 + 5d, n = 3; S7 + 5d, n = 7 and R7 + 5d, n = 4); two-way ANOVA, NS. (**C**) O_2_/CO_2_ rate (mean ± SEM) calculated from O_2_ and CO_2_ concentration in the oxygen consumption conditioned buffer from P1 and P7 sham and resected neonatal hearts 5 days after the procedure (S1 + 5d, n = 9 and R1 + 5d, n = 3; S7 + 5d, n = 7 and R7 + 5d, n = 4); two-way ANOVA, NS.

The neonatal mammalian heart can regenerate following injury, mainly through cardiomyocyte proliferation. We previously demonstrated that robust regeneration occurred after cardiac apex resection in P1 neonatal rats, with cardiomyocyte neoformation and preserved cardiac function, while P7 pups failed to display this regenerative capacity^2^. We then determined oxidative metabolism rates in cardiac tissue samples from resected animals. Interestingly, we found that P1 resected cardiac ventricle tissue consumed oxygen at faster rates in the presence of glucose even five days after the procedure compared to P7 animals (Fig. 5A). Oxygen consumption rates were equal between sham and resected tissues, indicating that the resection process itself was not responsible for the higher respiratory rates, but rather that these rates reflected the developmental stage of the newborn animals. Lactate levels were equal in all samples (Fig. 5B), indicating that fermentation did not change in this period, and O_2_/CO_2_ ratios were also unchanged, indicating maintenance of the proportion of oxidized lipid and carbohydrate (Fig. 5C).

**Figure 6:**
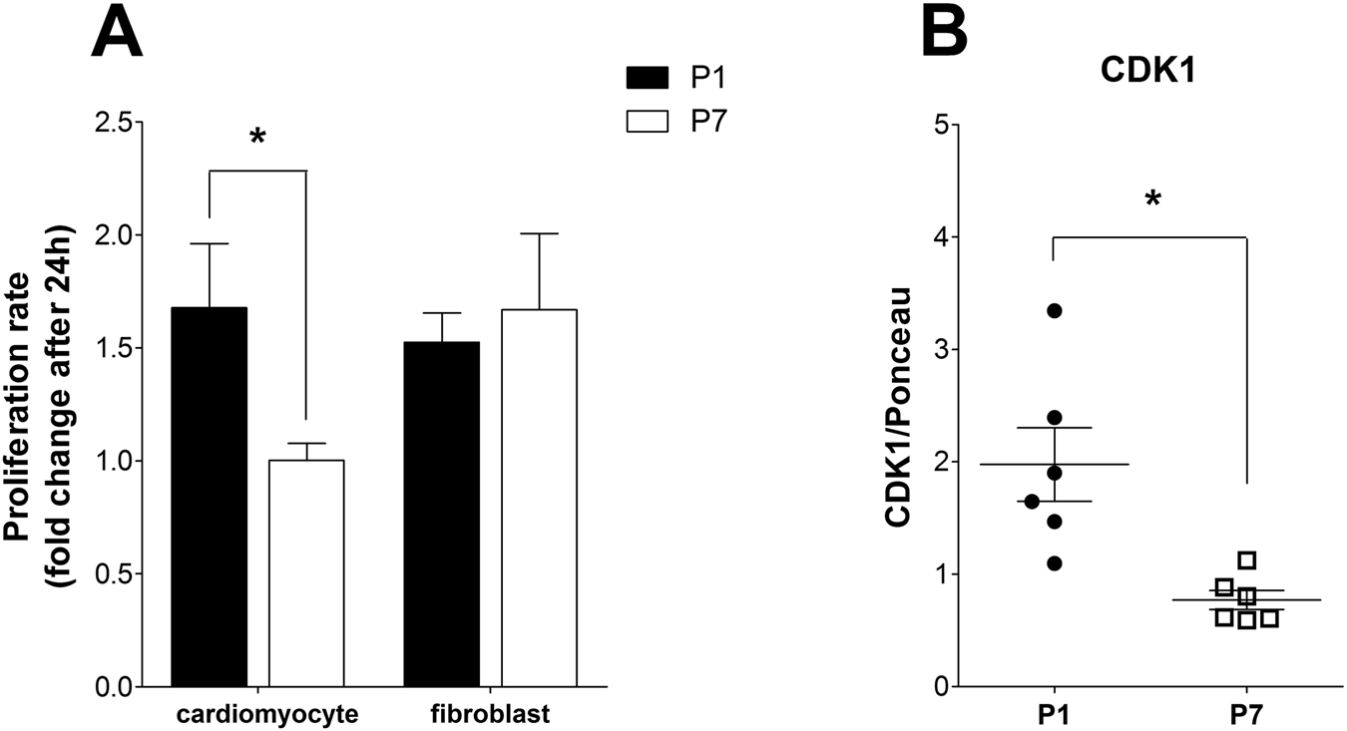
P1 cardiomyocytes maintain high proliferative rates in culture. (**A**) Bars indicate P1 (n = 7) and P7 (n = 4) cardiomyocyte and fibroblast proliferative rates (mean ± SEM) after 24 hours in culture; two-way ANOVA, *p < 0.05 vs. P1. (**B**) CDK1 protein expression (mean ± SD); t-test, *p < 0.05 vs. P1); t-test, *p < 0.05 vs. P1, in P1 and P7 hearts (n = 6).

These results suggest that high levels of oxidative metabolism support the proliferative activity required for cardiac regeneration that occurs only within a narrow postnatal period. To further explore these findings, we measured cell proliferation *in vitro* in P1 and P7 cardiomyocytes cultures using high-content screening assays for serum-induced proliferation. To accurately differentiate cardiomyocytes from fibroblasts in culture, we used monoclonal antibodies against sarcomeric tropomyosin and vimentin, respectively, in P1 and P7 cultures. Data are presented as proliferation rates *in vitro* after 24 hours of culture. The cardiomyocyte proliferation rate was significantly greater in P1 compared to P7 cultures (Fig. 6), confirming their enhanced proliferation in response to serum. Fibroblast proliferation rates were similar in P1 and P7 cultures (Fig. 6A). Corroborating this, CDK1 protein and cell cycle gene markers were higher in P1 heart tissues, indicating maintenance of cell cycle activity (Fig. 6B and Supp. Figure 2, respectively). CDK1 gene expression differences tended in the same direction (Supp. Figure 2; p = 0.0592).

**Figure 7:**
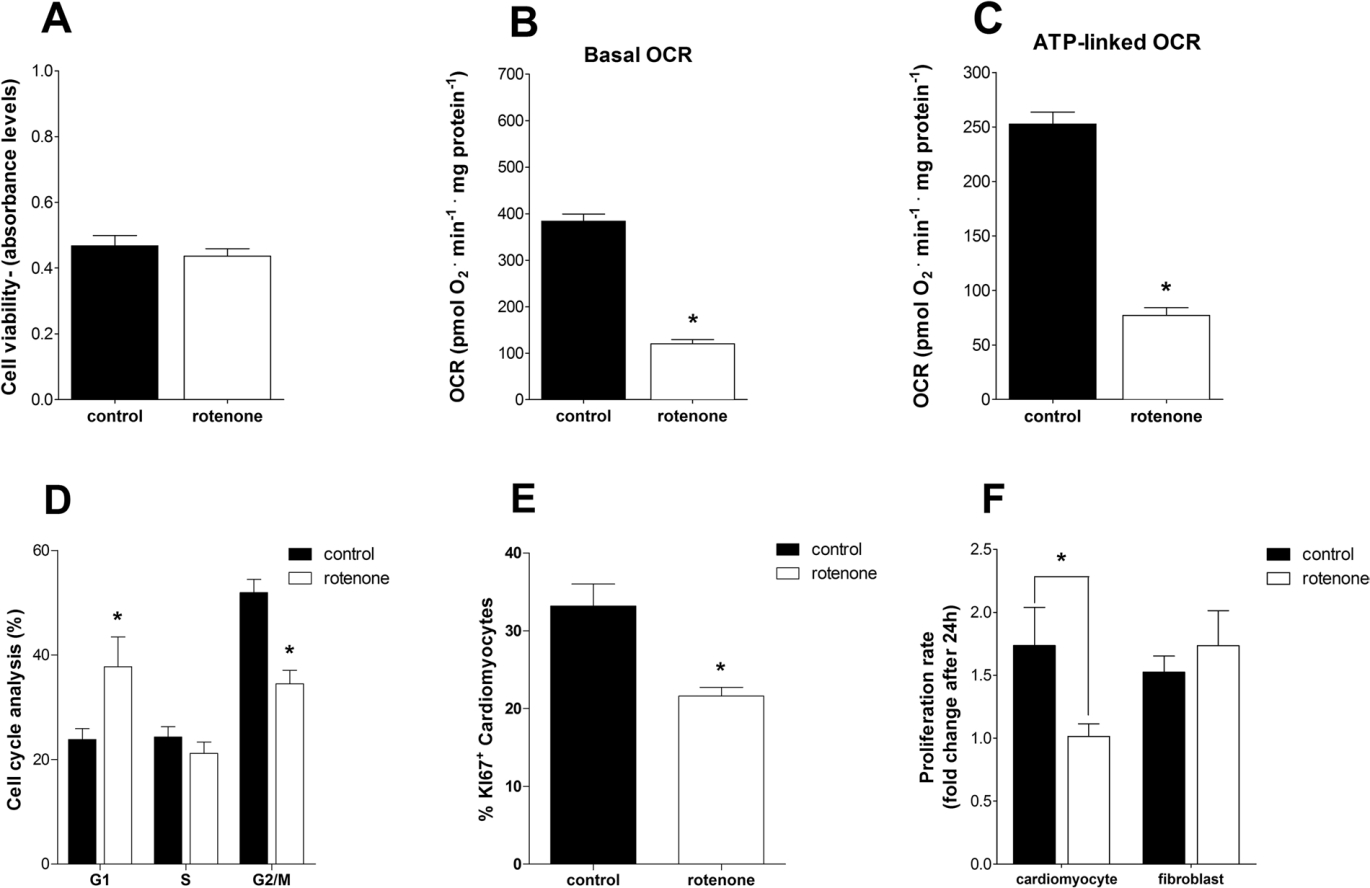
Decreased oxygen consumption reduces P1 cardiomyocyte proliferative rates. (**A**) Cell viability (mean ± SEM) measured in P1 cardiomyocyte culture under control conditions and treated for 48 hours with 5 nM rotenone (n = 3 for both); t-test, NS. (**B**) Basal OCR (mean ± SEM) using a Seahorse Flux Analyzer in P1 cardiomyocyte cultures, control condition and treated with 5 nM rotenone for 48 hours (n = 3 for both); t-test, *p < 0.05 vs. control. (**C**) ATP-linked OCR (mean ± SEM) in P1 cardiomyocyte cultures, control condition and treated with 5 nM of rotenone for 48 hours (n = 3 for both); t-test, *p < 0.05 vs. control. (**D**) Cell cycle analysis (mean ± SEM) in P1 cardiomyocyte cultures under control conditions and treated for 48 hours with 5 nM rotenone (n = 3 for both); two-way ANOVA, *p < 0.05 vs. control. (**E**) Percentage of Ki67^+^ cardiomyocytes (mean ± SEM) in P1 cardiomyocyte cultures (n = 4 for both); t-test, *p < 0.05). (**F**) Bars indicate P1 cardiomyocyte and fibroblast proliferative rates (mean ± SEM) of after 24 hours in culture under control conditions (n = 7) or when treated with 5 nM rotenone (n = 6) for 48 hours; two-way ANOVA, *p < 0.05 vs. control.

Next, we sought to test the contribution of oxidative metabolism to the proliferative capability of P1 cells by promoting a partial, nonlethal, chemical inhibition of respiration and following its effect on the proliferation of P1 cells. The mitochondrial complex I inhibitor rotenone, which promotes the accumulation of NADH, and thus inhibition of both the tricarboxylic acid cycle and β-oxidation, was used at a dose of 5 nM, which did not affect cell viability (Fig. 7A). This dose, however, reduced mitochondrial oxidative metabolism with a marked effect on ATP-linked oxygen consumption (Fig. 7B,C). Interestingly, the reduction in mitochondrial oxygen consumption was sufficient to reduce cell cycle activity, measured by differential condensed chromosomes and Ki67 expression (Fig. 7D and E, respectively), and to inhibit the proliferative capacity of P1 cardiomyocytes (Fig. 7F). Conversely, this seems not to affect fibroblast proliferation (Fig. 7F). Overall, these results show that high mitochondrial respiratory activity in P1 cells is required to sustain cardiomyocyte proliferation.

## Discussion

The main finding of the present work is that the early postnatal high cardiac metabolism and accompanying cell proliferation correlates with oxidative energy metabolism. The energy metabolism demand subsides as proliferation ceases and oxidative energy metabolism prevails as the main energetic source for the cardiac tissue after that.

Recent evidence indicates that rodent hearts display a regenerative capacity restricted to a period of few days after birth, after which cardiomyocytes permanently differentiate. This transition is complex, and data suggest a role for atmospheric oxygen tension as a trigger^13^. Also, one may hypothesize that the process involves a shift from more fermentative to more oxidative metabolism. Indeed, gene expression patterns, reported here (Fig. 1) and previously^22^, and quantitative mass spectrometry analysis^13^ show a striking increase in the expression of all energy metabolism pathways that require oxygen.

However, the gene expression pattern may be limited as a proxy of metabolic activity, since much of metabolic regulation involves post-translational modifications and allosteric mechanisms^15–17^. Indeed, our functional assessment of respiratory activity in neonatal pup hearts, using both tissue slices (Fig. 2A) and cultured cells (Fig. 4) shows that oxidative metabolism decreases between the first and seventh days after birth. This decrease involves an overall dampening in metabolic fluxes because it occurs in the presence of both glucose and fatty acids (Fig. 2A). A parallel decrease in lactate production is also observed (Fig. 2B), indicating that anaerobic glycolysis is also blunted during perinatal heart development, suggesting that overall energetic demands decrease in the days after birth. These effects are not accompanied by changes in mitochondrial mass (Fig. 3). Indeed, cardiomyocyte extracellular flux analysis experiments indicate that the decrease in oxidative metabolism between P1 and P7 was mostly related to lower rates of ATP-linked mitochondrial respiration (Fig. 4). Overall, these results indicate that gene expression analysis is a poor indicator for functional metabolic status and suggest that metabolic activity is the preferred indicator to be evaluated whenever possible. It also may apply to differentiating cells, in which significant changes in protein turnover may occur.

Our results indicate that a decrease in oxidative metabolism over the first days after birth accompanies postnatal cardiac maturation. Metabolic changes that occur during differentiation have been evaluated only sporadically in other tissues, and have varying results. Neuronal differentiation also involves a decrease in oxidative metabolism, but, differently from our findings with cardiomyocytes, with a concomitant increase in fermentation^23^. Mesenchymal stem cell differentiation to adipocytes and osteocytes involves increases in oxygen consumption rates, but differentiation into chondrocytes involves a large decrease in respiratory activity^9^. Overall, these studies show that there is no specific rule regarding oxidative metabolism and reproductive capacity, but rather that oxidative metabolic rates follow energetic necessities, which are variable during differentiation.

The mechanisms behind this shift from high oxidative metabolic rates to lower oxidative rates in neonatal cardiomyocytes are complex and remain to be determined. Recently, Puente *et al*.^13^ demonstrated that hypoxia depresses postnatal cardiomyocyte cell cycle arrest and mitochondrially-targeted antioxidants prevent it. Because the significant accumulation of markers of oxidative damage also occurs in this process, the authors concluded that redox signaling processes triggered by atmospheric air mediate cell cycle arrest. This finding raises the interesting possibility that redox signaling may also be responsible for the decrease in oxygen consumption that occurs between P1 and P7.

Irrespective of the mechanism whereby oxidative metabolism is dampened days after birth, we established that the maintenance of high electron transfer rates is pivotal for cardiomyocyte proliferation (Fig. 7), and therefore regeneration, consistent with the high energy expenditure required for cardiomyocyte proliferation. Primitive fetal liver hematopoietic stem cells use oxidative phosphorylation to generate ATP more efficiently for extensive cell expansion^8^. Recently, hypoxia and glucose deprivation was shown to reduce ATP levels in murine induced stem cell cardiomyocytes and neonatal cardiomyocytes, with an increase in oxidant production and cell death specially in the former^24^. Interestingly, our results show that the relationship between oxygen consumption and the ability to proliferate *in vitro* is restricted to cardiomyocytes.

Overall, our results using functional techniques demonstrate that a marked decrease in oxygen-dependent metabolic rates occurs during postnatal cardiac development and that the high respiratory rates present in neonatal cells are necessary for cardiomyocyte proliferation. Based on these results, it is tempting to speculate that interventions designed to extend the narrow “regenerative window” of newborn hearts may include a coadjutant stimulus to promote mitochondrial biogenesis or decrease mitophagy^25^ and may also be relevant for strategies aimed at expanding cardiac cells *in vitro* for transplantation or stimulation of endogenous cardiac proliferation therapies.

## Methods

### Animals

We used in this study Wistar rats in postnatal day 1 (P1) and postnatal day 7 (P7). They were maintained with the mother rat in a 12:12 hour light–dark cycle and temperature-controlled environment (22 °C) with free access to food and water.

The Institutional Review Board of the University of São Paulo Medical School, Brazil aprooved the experimental procedures (#285/12), which followed the US National Institutes of Health and institutional guidelines for care and use of laboratory animals.

### Cardiomyocyte isolation and culture

We euthanized the P1 and P7 neonatal rats by decapitation, and the hearts were excised and rinsed in cold ADS buffer. We pooled a total of 8–10 neonatal hearts in each isolation. We removed the atria and minced and digested the ventricles in 3 mL of pre-warmed digestion buffer containing collagenase II (Worthington Chemicals) and pancreatin (Sigma-Aldrich) in a shaking incubator set at 37 °C for 15 minutes. After this, we transferred the digested material to 15 mL tubes for inactivation with media containing two mL Dulbecco’s Modified Eagle’s Medium and M199 (4:1) containing 10% Donor Horse Serum, 5% Newborn Calf Serum, and 1% penicillin/streptomycin (Invitrogen, Life Technologies). We centriguged the supernatant collected from each digest at 220 × g for 5 minutes, and the cell pellet was resuspended in fresh medium and maintained at 37 °C. We repeated six times the incubation and centrifugation steps and resuspended the final cell pellet from all digests in a 100-µm cell strainer, followed by two mL of fresh media. Adherent cells were attached for 45 minutes in culture dishes at 37 °C in a humidified atmosphere of 95% air and 5% CO_2_. The non-adherent fraction (myocytes) was centrifuged at 220 × g for 5 minutes and counted in a Neubauer chamber. We plated the cells on laminin-coated plates. After 48 hours, cardiomyocytes formed a monolayer of viable and spontaneously beating cells, and they were trypsinized and frozen for later use.

### Tissue oxygen consumption assays

Mitochondrial oxygen consumption was monitored using a computer-interfaced Clark-type electrode, as previously described^18^, with continuous stirring at 37 °C. For cardiac ventricle tissue, oxygen consumption assays were performed using Oroboros O2k high-resolution respirometry (Oroboros Instruments, Bioblast). We added each cardiac ventricle slice (1–2 mm) into the equipment chamber with two mL of Krebs buffer without substrates, which allowed the maintenance of the physical structure of the thin neonatal cardiac tissue. After recording basal respiration, we added glucose (5 mM) or oleic acid (100 µM) and registerd the oxygen consumption rate for a further 5 minutes. We normalized the results by basal respiration and tissue protein content. We stored conditioned buffer and heart tissue for future analysis.

### Seahorse oxygen consumption rate (OCR) measurements

We measured oxygen consumption rates (OCR) in P1 and P7 primary cardiomyocyte cultures using an XF24 Extracellular Analyzer (extracellular flux, 24-well plate, Seahorse Bioscience), as previously described^19,20^ and also exposed P1 cardiomyocytes to 5 nM rotenone (Sigma-Aldrich). The cells were seeded as three or four replicates in a laminin-coated XF24 24-well culture microplate at 40,000 cells/well (0.21 cm^2^ growth area) in 500 μL of growth medium and incubated for 48 hours at 37 °C in a humidified atmosphere of 95% air and 5% CO_2_. Before the assay, the medium was removed and replaced by 500 μL of bicarbonate-free assay medium (114 mM NaCl, 4.7 mM KCl, 1.2 mM KH_2_PO_4_, 1.16 mM MgSO_4_, 2.5 mM CaCl_2_, pH 7.2, and 2.8 mM glucose). The cells were pre-incubated for 1 hour at 37 °C in air. We determined respiration-driven ATP synthesis and proton leak-driven respiration by the addition of oligomycin (4 μg/mL). After three measurement cycles, five μM of the uncoupler carbonyl cyanide-p-trifluoromethoxy-phenylhydrazone (CCCP) was added to determine maximal respiratory capacity. After a further three measurement cycles, one μM rotenone was added to block complex I in addition to 1 μM antimycin A to inhibit complex III, thereby ablating mitochondrial oxygen consumption. We normalized OCR by the amount of cellular protein content in each well.

### Lactate production and O_2_/CO_2_ ratios

Conditioned buffers from tissue oxygen consumption assays were used to measure lactate concentrations, diluted oxygen, and dioxide carbon concentrations in duplicate, using *ABL 800 Flex* equipment, according to the manufacturer’s instructions. We used protein content to normalize the measures.

### Enzymatic activities

Enzymatic activities were determined in cardiac ventricle tissues using kinetic assays. For hexokinase activity, we homogenized the heart samples in buffer containing 50 mM of Trietanolamin, 50 mM Tris-HCl, one mM EDTA, two mM of MgCl_2_, and 30 mM β-mercaptoethanol (pH 7.5). The suspension was centrifuged and the resulting supernatant was added in duplicate to a 96-well plate with 75 mM Tris-HCl, 0.8 mM EDTA, 7.5 mM MgCl_2_, 1.5 mM KCl, and 4 mM β-mercaptoethanol (pH 7.5), containing 0.4 mM NADP^+^, ATP (1.8 mM), creatine phosphokinase (1.8 U), phosphocreatine (0.4 mM), 1% TRITON-X 100, and glucose-6-phosphate dehydrogenase. The time scan started after the addition of glucose, and the activity was estimated using a spectrophotometer at 340 nm. For lactate dehydrogenase activity, we homogeneized the heart samples in buffer containing 77 mM of Tris-HCl, one mM EDTA, two mM MgCl_2_, and four mM β-mercaptoethanol (pH 7.5). We centrifuged the suspension, and the added the resulting supernatant in duplicate to a 96-well plate with 120 mM Tris-HCl, 3.4 mM NADH, and 1% TRITON X-100 (pH 7.5). We began the the time scan by adding 20 mM of pyruvate and estimated the activity following the absorbance at 340 nm. For citrate synthase activity, we homogenized the heart samples in buffer containing 50 mM Tris and one mM EDTA (pH 7.4) and centrifuged the suspension and added the resulting supernatant in duplicate to a 96-well plate with 100 mM Tris, 0.2 mM DTNB, 30 mM acetyl-coenzyme A and 1% TRITON X-100 (pH 8.1). We began the time scan by adding 0.5 mM oxaloacetic acid, and estimated the activity following the absorbance at 412 nm.

### Cell viability

P1 cardiomyocyte culture viability was determined using the MTT assay. Cells were plated in duplicate at 20,000 cells/well in 96-well plates and maintained in serum-free medium for the measurement. We added 20 µL of MTT solution (Thiazolyl Blue Tetrazolium Bromide, Sigma-Aldrich) at 5 mg/mL in PBS to each well after 45 hours of treatment with 5 nM rotenone. The plate was shaken and incubated again at 37 °C for 3 hours. We removed the culture medium and added 200 µL of DMSO and performed the reading at 560 nm. Absorbance measured was directly related to cell viability.

### Apical resection surgery

P1 and P7 neonatal rats underwent heart apex resection as previously described^2^. Neonates were anesthetized by hypothermia for 10 minutes and kept in the inhaler with isoflurane during the procedure (Isoforine - Cristália). After a small skin incision, we performed a ventrolateral thoracotomy at the third intercostal space by dissection of the intercostal muscles. The heart was exposed, and the apex was resected using iridectomy scissors. We closed the thoracic wall incision using 7.0 non-absorbable silk sutures. For recovery, the neonates were placed on a warmed plate (29 °C) under a heat lamp with oxygen flow until fully conscious. In Sham surgery, we did not perform the resection. We euthaneized the P1, P7, and sham rats by decapitation five days after apical resection and collected the hearts.

### High content screening cardiomyocyte proliferation rates

We seeded P1 and P7 primary cardiomyocytes in duplicate on laminin-coated 96-well plates at 10,000 cells/well in Dulbecco’s Modified Eagle’s Medium and M199 (4:1) containing 10% Donor Horse Serum, 5% Newborn Calf Serum, and 1% penicillin/streptomycin (Invitrogen, Life Technologies), and incubated at 37 °C in a humidified atmosphere of 95% air and 5% CO_2_ for 24 and 48 hours. We also exposed P1 cardiomyocytes to 5 nM rotenone (Sigma-Aldrich). After each time point, for imaging, we incubated the cells for 30 min at 37 °C in the presence of Hoechst 33342 (1 µg) and the membrane potential-sensitive fluorescent dye tetramethylrhodamine methyl ester (TMRM, 15 nM, Invitrogen, Life Technologies). After the live acquisition, cells were fixed with 4% paraformaldehyde, permeabilized with 0.1% Triton X-100 for 15 minutes at 4 °C, and blocked with 5% serum albumin bovine for 60 minutes at room temperature. For immunostaining, anti-tropomyosin (sarcomeric) antibody (T9283 – 1:200, Sigma-Aldrich), anti-vimentin antibody (ab92547 – 1:300, Abcam) and anti-Ki67 antibody (ab16667 - 1:300, Abcam) were added and incubated overnight at 4°C, followed by washing. Secondary antibodies (Alexa 488 and 555–1:500, Molecular Probes, Invitrogen, Life Technologies) were added and incubated for 1 hour at room temperature, followed by washing. We labeled cell nuclei with DAPI nuclear stain (1:100). Images were acquired with an IN Cell Analyzer 2200 high-content imaging system (GE Healthcare, USA) configured with the standard size CMOS camera. Images of live cells were acquired using 40x (0.6NA) objective (9 images per well-brightfield, Hoechst 33342, Cy3); images of fixed cells were acquired using 20x (0.45 NA) objective (20 images per well-brightfield, DAPI, FITC, Cy3). Analysis protocols were developed with IN Cell Investigator^TM^ software (CM and fibroblasts differential counting GE Healthcare, USA) as well as using a python script (for %KI67^+^ cardiomyocytes enumeration). The area of functional mitochondria was quantified using the cell-integrated area of Cy3 channel to investigate TMRM-stained area (in pixels). Cardiomyocytes and fibroblasts were identified, differentiated, and quantified using the cell-integrated intensity of the FITC channel for Tropomyosin positive cells and Cy3 channel for Vimentin positive cells. We estimated the proliferation rate as the ratio between the number of cells after 48 hours in culture normalized to the number of cells after 24 hours in culture. The percentage of Ki67 + cardiomyocytes was obtained by counting and summing all cardiomyocytes (defined as tropomyosin^+^ cells, FITC channel) with or without KI67 staining in the nucleus for every 20 images per well. The average of %KI67 + cardiomyocytes per well is reported. We analyzed cell cycle phases by differential total DAPI signal per nucleus and categorized into G1, S and G2/M cell cycle phases. We used a function in the Spotifre Decision Site data visualization software (Tibco) provided along with IN Cell Investigator software.

### RNASeq analysis

We prepared the libraries, and next-generation sequencing - RNASeq (also known as WTSS – Whole-transcriptome Shotgun Sequencing) in the “Large-Scale Sequencing Laboratory” facility located at the University of São Paulo Medical School, FMUSP, São Paulo-SP, Brazil. Total RNA from samples was purified (RNeasy Mini Kit, Qiagen) and analyzed with the Agilent 2100 Bioanalyzer (Agilent Technologies). Samples with RIN higher than 9.0 and A260/280 higher than 1.8 were considered adequate for library preparation with TruSeq Stranded Total RNA with “Ribo-Zero” Gold kit (Illumina). Libraries were individually quantified through real-time PCR (qPCR) and sequenced with HiSeq. 2500 equipment (Illumina) according to the manufacturer’s instructions with pair-end reads (2 × 100 cycles). Data were analyzed using bioinformatics tools. We estimated differential gene expression with the DESEq v1.12.1 package using the R-Bioconductor. Annotations of genes were made using Biomart (R-Bioconductor) software.

### Western blots

We denatured tissue lysate proteins (40 µg) by heating at 100 °C for 5 minutes; separeted the proteins using SDS-PAGE and transferred to PVDF membranes (Amersham Hybond^TM^–P, GE Healthcare). We blocked the blotted membranes with 5% bovine serum albumin overnight at 4 °C. We detected individual proteins by blotting with specific primary antibodies for 1 hour at room temperature using anti-VDAC (ab15895, 1:1000, Abcam), anti-COX IV (#4840, 1:1000, Cell Signaling), anti-CDK1 (ab32384, 1:1000, Abcam), anti-LC3 (#2775, 1:1000, Cell Signaling) and anti-beta-actin (ab8227, 1:2000, Abcam) followed by secondary probing with HRP-conjugated (1:1000 dilution). Immunodetection was determined using the enhanced chemiluminescence (ECL) method. We used ImageJ software (NIH software) for densitometry normalized it with beta-actin expression.

### Gene expression measurements by RT-PCR and mitochondrial DNA quantification

We isolated total RNA using PureLinK^TM^ RNA Mini Kit (Life Technologies, USA) following the manufacturer’s instructions and performed cDNA synthesis with a SuperScript^TM^ III First-Strand Synthesis System for RT-PCR kit (Invitrogen, Life Technologies). Reverse transcription polymerase chain reaction (RT-PCR) was performed in duplicate with a QuantStudio^TM^ 12 K Flex Real-Time PCR System (MicroAmp® Optical 384-well Reaction Plate), using Taq polymerase SYBR® Green PCR Master Mix (Applied Biosystem, Foster City, CA) under the following conditions: initial denaturation at 95 °C for 10 minutes; 40 cycles of denaturation at 95 °C for 15 seconds, annealing and extension at 60 °C for 1 minute; final extension at 72 °C for 5 minutes. The fluorescent signal was collected at the end of every cycle. Data are presented as 2^−∆∆Ct^, with ∆∆Ct = ∆Ct_experimental_ − ∆Ct_control_ and standard deviation was calculated using the following formula: $s^{\prime} =\sqrt{{s}_{{experimental}}^{2}+{s}_{{control}}^{2}}$ when s is the standard deviation of the corresponding delta-ct’s on dataset. For mitochondrial DNA relative quantification qPCR, Mterf1 and mitochondrial Cox1 genes was assayed in based on protocol previously described^26^. Primers sequences and details are presented on Supplemental Table 1.

### Statistical analysis

Results are presented as means ± standard error of the mean. The unpaired Student *t* test and two-way ANOVA with post hoc Bonferroni’s test were used to compare groups as appropriate. RT-PCR and mitochondrial DNA normalized by genomic DNA t-tests were analyzed using the ∆Ct_experimental_ dataset. All statistical analyses were performed using GraphPad Prism 5.0 (GraphPad Softwares Inc., CA, USA). A p ≤ 0.05 value was considered statistically significant.

## Electronic supplementary material

Supplementary Information

## References

[1] Porrello E (2011). Transient regenerative potential of the neonatal mouse heart. Science.

[2] Zogbi C, Saturi de Carvalho AE (2014). Early postnatal rat ventricle resection leads to long-term preserved cardiac function despite tissue hypoperfusion. Physiological Reports.

[3] Alkass K (2015). No evidence for cardiomyocyte number expansion in preadolescent mice. Cell.

[4] Senyo SE (2013). Mammalian heart renewal by pre-existing cardiomyocytes. Nature.

[5] Bergmann O (2009). Evidence for cardiomyocyte renewal in humans. Science.

[6] Prosdocimo G, Giacca M (2017). Manipulating the proliferative potential of cardiomyocytes by gene transfer. Methods Mol Biol.

[7] Leone M, Magadum A, Engel FB (2015). Cardiomyocyte proliferation in cardiac development and regeneration: a guide to methodologies and interpretations. Am J Physiol Heart Circ Physiol.

[8] Manesia JK (2015). Highly proliferative primitive fetal liver hematopoietic stem cells are fueled by oxidative metabolic pathways. Stem Cell Research.

[9] Forni MF, Peloggia J, Trudeau K, Shirihai O, Kowaltowski AJ (2015). Murine mesenchymal stem cell commitment to differentiation is regulated by mitochondrial dynamics. Stem Cell.

[10] Lopaschuk G, Jaswal JS (2010). Energy metabolic phenotype of the cardiomyocyte during development, differentiation, and postnatal maturation. J Cardiovasc Pharmacol.

[11] Lopaschuk G, Spafford MA, Marsh DR (1991). Glycolysis is the predominant source of myocardial ATP production immediately after birth. Am J Physiol.

[12] Lai L (2008). Transcriptional coactivators PGC-1alpha and PGC-lbeta control overlapping programs required for perinatal maturation of the heart. Genes Dev.

[13] Puente B (2014). The oxygen-rich postnatal environment induces cardiomyocyte cell-cycle arrest through DNA damage response. Cell.

[14] Neary MT (2014). Hypoxia signaling controls postnatal changes in cardiac mitochondrial morphology and function. J Mol Cell Cardiol.

[15] Mendler L, Braun T, Muller S (2016). The Ubiquitin-Like SUMO System and Heart Function: From Development to Disease. Circ Res.

[16] Wadosky KM, Willis MS (2012). The story so far: post-translational regulation of peroxisome proliferator-activated receptors by ubiquitination and SUMOylation. Am J Physiol Heart Circ Physiol.

[17] Lai L (2014). Energy metabolic reprogramming in the hypertrophied and early stage failing heart: a multisystems approach. Circ Heart Fail.

[18] Hütter E, Unterluggauer H, Garedew A, Jansen-Dürr P, Gnaiger E (2006). High-resolution respirometry–a modern tool in aging research. Exp Gerontol.

[19] Brand MD, Nicholls DG (2011). Assessing mitochondrial dysfunction in cells. Biochem J.

[20] Nicholls, D.G. *et al*. Bioenergetic profile experiment using C2C12 myoblast cells. *Journal of visualized experiments - JoVE* **46** (2010). 10.3791/2511 PMC3159644 21189469

[21] Lemasters JJ (2005). Selective mitochondrial autophagy, or mitophagy, as a targeted defense against oxidative stress, mitochondrial dysfunction, and aging. Rejuvenation Res.

[22] Pohjoismaki J (2012). Oxidative stress during mitochondrial biogenesis compromises mtDNA integrity in growing hearts and induces a global DNA repair response. Nucleic Acids Res.

[23] Fornazari M (2011). Neuronal differentiation involves a shift from glucose oxidation to fermentation. J Bioenerg Biomembr.

[24] Brodarac A (2015). Susceptibility of murine induced pluripotent stem cell-derived cardiomyocytes to hypoxia and nutrient deprivation. Stem Cell Res Ther.

[25] Delbridge LM, Mellor KM, Taylor DJ, Gottlieb RA (2015). Myocardial autophagic energy stress responses—macroautophagy, mitophagy, and glycophagy. Am J Physiol Heart Circ Physiol.

[26] Rooney JP (2015). PCR based determination of mitochondrial DNA copy number in multiple species. MethodS Mol Biol.

