# Supplementary material for: Early Postnatal Cardiomyocyte Proliferation Requires High Oxidative Energy Metabolism
Source: Sci Rep. 2017 Nov 13;7:15434. doi: 10.1038/s41598-017-15656-3 (PMC5684334; doi:10.1038/s41598-017-15656-3)
Supplement: Supplementary file 1 — Supplementary Information [file 41598_2017_15656_MOESM1_ESM.pdf]

Early Postnatal Cardiomyocyte Proliferation Requires High Oxidative Energy Metabolism

Ana Elisa Teófilo Saturi de Carvalho[1], Vinícius Bassaneze[1], Maria Fernanda Forni[2], Aline Alfonso Keusseyan[1], Alicia Juliana Kowaltowski[2], José Eduardo Krieger[1] *

[1] Laboratory of Genetics and Molecular Cardiology/LIM 13, Heart Institute (InCor- HCFMUSP), University of São Paulo Medical School, São Paulo, Brazil.

[2] Departamento de Bioquímica, Instituto de Química, Universidade de São Paulo, Brazil.

* Correspondence to Jose E. Krieger, Genetics and Molecular Cardiology/LIM 13, Heart Institute, University of São Paulo Medical School, Av. Dr. Enéas C. Aguiar 44, 05403-000, São Paulo, SP, Brazil. http://genetica.incor.usp.br/

Telephone: 55 11 3069 5068; Fax: 55 11 3069 5022

E-mail: krieger@incor.usp.br

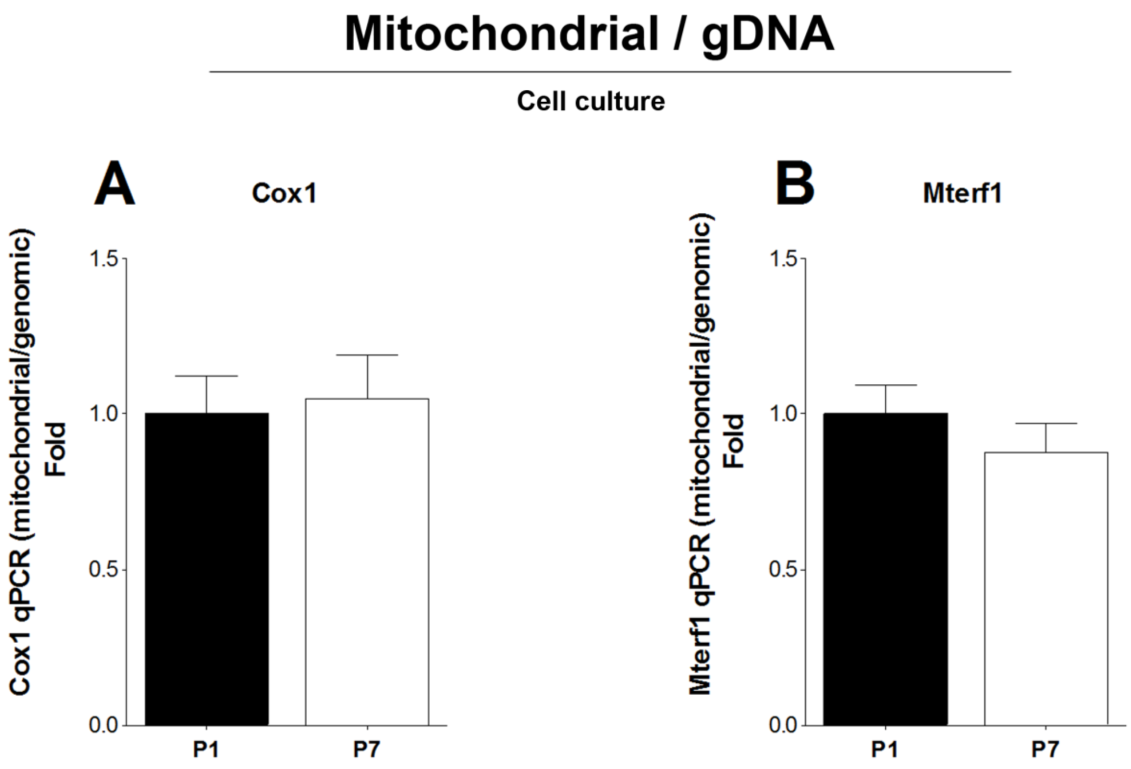

Supplemental Figure 1: Mitochondrial DNA quantification for Cox I and Mterf1 genes as a measure of mitochondrial content in cell cultures (P1 vs. P7; (n = 6 for both); t-test, NS). Data is normalized to genomic DNA.

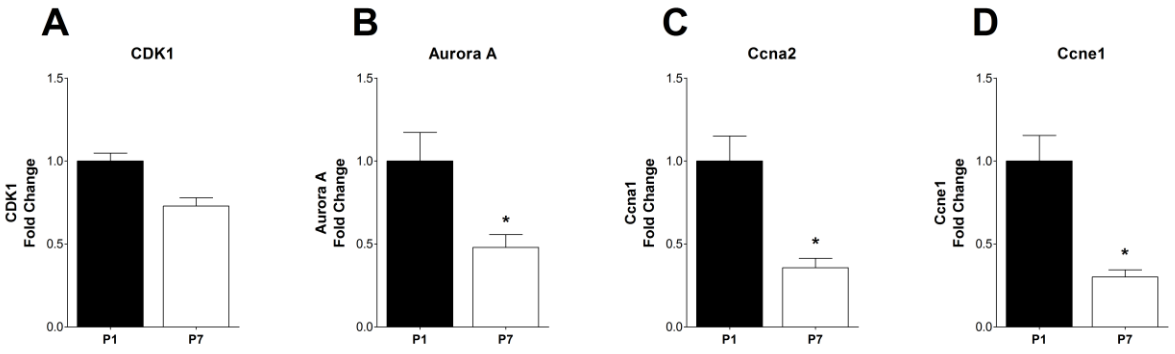

Supplemental Figure 2: Expression profile of genes related to proliferation in P1 vs. P7 tissues. A) Cdk1, B) Aurora A, C) Ccna2 and D) Ccne1. Fold change ± SD, * p<0.05).

Supplemental Table 1: List of all primers used in this study. For primers used in RT-PCR experiments, at least one intron between each pair of primers was considered to avoid genomic DNA amplification.

Primer	RefSeq ID	Primer Sequence	Tm (ºC)	Amplicon Size
r Aldoc Fw	NM_012497.1	GAATGGTATCGTGCCCATCG	60	211
r Aldoc Rev		GGCGATCTCTTCTGGGCTAT		
r Lrrk2 Fw	NM_001191789.1	AGCTGTCCTACTCTGGGAGG	60	204
r Lrrk2 Rev		ACCCCAAAACCAGCATGACA		
r Ndufv3 Fw	NM_001101011.2	TCTCGAGGCAAAGGTGTTCC	60	157
r Ndufv3 Rev		GTAGGTGGTGGTGTCACTCG		
r Ucp2 Fw	NM_019354.3	CTTGATCTTGGAGGCCTCAGC	60	138
r Ucp2 Rev		TCTGACTTCCTGCTACCTCCC		
r Ccne1 Fw	NM_001100821.1	CCTCTCAGTCCGATCCAGAAA	60	172
r Ccne1 Rev		TTTGTTAGGGGTGGGGATGA		
r Ccna2 Fw	NM_053702.3	CCTGCATTTGGCTGTGAACT	60	141
r Ccna2 Rev		AACTCTGCTACTTCTGGGGG		
r Aurka Fw	NM_153296.2	GTATTTCCATGACGCCACCA	60	140
r Aurka Rev		ACAAAGCATTCGCCAACTCC		
r Actb Fw	NM_031144.3	TCTACAATGAGCTGCGTGTG	60	76
r Actb Rev		TTGGCCTTAGGGTTCAGAGG		
r Cox1 Fw	NC_001665.2	TGCTGGAGCATCCGTAGATT	60	63
r Cox1 Rev		AAGATAGAAGACACCCCGGC		
r Mterf1 Fw	NC_005103.4	CTGCTCTGTTGGGTTGACTC	60	75
r Mterf1 Rev		AGACTGTTGGAGAAGGTCCTG		
